# The relationship between social support and employment anxiety in preschool teacher trainees in China: the mediating role of psychological capital and the moderating role of professional identity

**DOI:** 10.3389/fpsyg.2025.1633440

**Published:** 2025-08-18

**Authors:** Li Li, Guanghua Wang, Meng Zhang

**Affiliations:** ^1^College of Teacher Education, East China Normal University, Shanghai, China; ^2^College of Education, Shanghai Normal University Tianhua College, Shanghai, China; ^3^International Collaborative Programs and Services, Shanghai Normal University Tianhua College, Shanghai, China

**Keywords:** preschool teacher trainees, social support, employment anxiety, psychological capital, professional identity

## Abstract

**Introduction:**

Against the backdrop of a continuously declining birth rate in China, pre-school teacher trainees are facing severe employment challenges and have experienced problems such as employment anxiety. Effective social support, positive psychological capital, and a high level of professional identity can act as a buffer against employment anxiety, alleviating the negative impact of the tense job market on employment anxiety.

**Methods:**

This study recruited 1195 students majoring in preschool education from Years 1 to 4 at four applied-oriented undergraduate institutions in Shanghai, China. The aim was to explore the influence of social support on the employment anxiety of preschool teacher trainees, and to analyze the mechanism of professional identity and psychological capital.These findings carry important theoretical and practical implications for alleviating preschool teacher trainees’ employment anxiety and enhancing their employment quality.

**Results:**

Research shows that (1) There are some significant differences in social support, psychological capital, employment anxiety and professional identity in terms of gender, grade and major choice; (2) social support negatively predicts employment anxiety; (3) psychological capital partially mediates the link between social support and employment anxiety; (4) professional identity significantly moderates the relationship between psychological capital and employment anxiety.

**Discussion:**

These findings carry important theoretical and practical implications for alleviating preschool teacher trainees’ employment anxiety and enhancing their employment quality.

## 1 Introduction

According to the Ministry of Education’s public data, by 2025, the demand for full-time kindergarten teachers will shrink significantly. Calculated on a per-class basis of 30 students, the demand will decrease by 38.1% compared to 2022. With a class size of 25, the drop will be at least 25.8%. Overall, since 2024, the supply of national and urban preschool education teachers has begun to surpass demand, with the surplus expected to keep growing (Hai and Gao, 2023). Intensifying competition in the labor market, ambiguous career prospects (Hu, 2024), and adaptive pressures associated with environmental transitions (Almarashdi, 2024) have emerged as the principal determinants precipitating employment-related anxiety among university students. According to social support theory, when individuals face difficulties and setbacks, an effective social support system can enhance their mental health and reduce negative emotions like anxiety and depression (Porter and Chambless, 2017). Studies reveal that the better the social support, the lower the level of employment-related anxiety. Good and sufficient social support can help individuals eliminate or alleviate the impact of stress on mental health, enhance their positive expectations, and effectively deal with social stress and setbacks (Wu, 2016). Multi-level support—emanating from society (e.g., employment facilitation and career guidance provided by governments or employers), educational institutions (e.g., structured practical-training programs), and the family (e.g., material and emotional support)—has been associated with significant reductions in university students’ employment-related anxiety and the preservation of their psychological well-being (Cheng, 2020; Guo and Li, 2025).

The external environment’s influence on individual behavior is mediated by psychological states (Li and Liu, 2020). When individuals subjectively appraise external support, they internalize it into their own emotion and cognition; this internal transformation process—conceptualized as perceived social support—subsequently guides their behavioral performance (Bao et al., 2022). Research indicates that internal factors, such as self-efficacy (Gao et al., 2020) and psychological resilience (Gao et al., 2021), can effectively predict individuals’ employment-related anxiety. In addition, evidence suggests that some factors of professional identity can predict the level of psychological capital (Yang and Han, 2016). The higher the students’ professional identity is, the lower their employment-related stress is Song et al. (2024), which is more conducive to alleviating anxiety (Gao and Zhang, 2019).

Previous studies on employment anxiety often focus on stress-coping strategies and psychological adjustments, rarely examining both external factors and internal psychological states. Meanwhile, few such studies center on preschool teacher trainees. Against this background, this study explores the relationships between social support, psychological capital, professional identity, and employment anxiety among preschool teacher trainees. In a sluggish job market context, the research aims to alleviate college students’ employment anxiety and improve their mental health, both of which are of great practical importance.

### 1.1 Social support and employment anxiety

Employment anxiety is a tense, restless, intense, and enduring emotional experience that individuals undergo when facing career choices, resulting in corresponding physiological and behavioral changes (Zhang and Chen, 2006). While moderate employment anxiety can stimulate individuals’ internal motivation for employment, enhancing their enthusiasm and perseverance in facing employment competition, excessive employment anxiety can lead to negative cognitive biases in employment, a decline in self - confidence, and may even trigger psychological discomfort such as avoidance and fear (Zhang, 2004); furthermore, it can impair individuals’ emotional intelligence (Ramos-Galarza et al., 2024). As the reserve force for future preschool education teachers, preschool teacher trainees confront significant employment challenges in the face of a drastically reduced job market, making them prone to severe employment anxiety that impacts their personal career development.

Social support, as a supportive resource, plays a crucial role in maintaining and sustaining positive emotional states. A good social support system is an important indicator of the level and quality of individual social interaction (Liu and Huang, 2010). The main effect theory of social support suggests that increasing social support can improve an individual’s mental health regardless of the initial level of social support they have (Chen et al., 2020). The buffering model of social support posits that social support protects individuals, acting as a buffer against negative emotions. Especially under external stress, it can mitigate the adverse effects of stress on physical and mental health (Klineberg et al., 2006). Existing research shows that social support, a key resource for individuals coping with stress, can alleviate employment pressure and enhance psychological resilience among graduates to some extent (Lin et al., 2004). Based on this, this study posits the following research hypothesis:

*H1*: Social support among preschool teacher trainees is a significant negative predictor of employment anxiety.

### 1.2 The mediating role of psychological capital

Conservation of resources (COR) theory posits that, driven by self-protection mechanisms, individuals seek to acquire, maintain, and create survival-enhancing resources. Resource depletion is seen as a major stress source, prompting individuals to actively build resources to successfully prevent and cope with actual or potential stressors and threats (Hobfoll, 2002). COR theory classifies resources into four categories. First are material resources, which are directly linked to socioeconomic status and are key to stress resistance, including items like cars and housing. Second are conditional resources that can facilitate individuals’ access to crucial resources, determining their stress-resistance potential, such as friends and power. Third are personality traits, particularly positive ones, which are essential for internal stress resistance, like self-efficacy and self-esteem. Fourth are energetic resources that help obtain the other three, such as time, money, and knowledge. Thus, social support, work development opportunities, optimistic personalities, autonomy, etc., can be deemed valuable resources by individuals (Cao and Qu, 2014). Psychological capital is the capital form of psychological resources (Cao and Qu, 2014). It refers to a positive psychological state formed during individual growth and development (Luthans et al., 2004). It is specifically manifested as confidence and the ability to exert necessary effort when facing challenging work in order to achieve success; making positive attributions for current and future success (optimism); persisting, quickly recovering, and adopting indirect paths to succeed when encountering problems and difficulties (resilience); and tenaciously pursuing goals, adjusting strategies when needed (hope) (Luthans et al., 2007). Individuals with high psychological capital can actively cope in uncertain and stressful environments (Zhang et al., 2019), and positively reframe negative events such as employment anxiety to reduce anxiety levels (Shi et al., 2023). Conversely, individuals with low psychological capital tend to report diminished sense of self-value, which impedes task initiation (Tran et al., 2024). In addition, research on social support and psychological capital indicates that social support is a key external protective factor influencing individual positive psychological levels (Zhu and Tao, 2022). High social support can meet individuals’ basic psychological needs and helps them experience positive emotions (Ibarra-Rovillard and Kuiper, 2011). Social support can reduce psychological stress when coping with life stressors and help individuals make more appropriate decisions (Huang et al., 2021).

Based on this, this study posits the following research hypothesis:

*H2*: Psychological capital mediates the relationship between social support and employment anxiety.

### 1.3 The moderating role of professional identity

According to self-identity theory, identity is individuals’ reflective comprehension of their existence’s meaning and value. It encompasses a profound comprehension of one’s identity, values, and pursuits and is a subjective inner experience (Dang, 2020). Professional identity, a specific manifestation of identity, refers to learners’ emotional acceptance and recognition of their discipline, based on their cognitive understanding, accompanied by positive external behaviors and inner appropriateness, and is a process of emotional, attitudinal, and cognitive internalization (Pan, 2009). Professional identity is both an existing state and a dynamic developmental process. Research shows that professional identity in college students is significantly positively correlated with psychological capital (Chen, 2016; Yang and Han, 2016), and professional identity significantly impacts psychological capital (Lin, 2021).

Some studies have also indicated that professional identity can significantly negatively predict employment anxiety (Hua, 2013; Wang and Wei, 2025). Based on this, this study posits that professional identity in preschool teacher trainees is a key moderator in psychological capital’s alleviation of employment anxiety. When preschool teacher trainees have higher levels of professional identity, they are more confident in their professional value, have stronger psychological capital, and are better able to alleviate employment anxiety. Conversely, lower professional identity can lead to doubts about their major studies, weakened psychological capital, and increased anxiety. Thus, this study proposes the following hypothesis:

*H3*: Professional identity positively moderates the relationship between psychological capital and employment anxiety.

### 1.4 Demographic differences

Demographic characteristics are primary determinants of social support, psychological capital, employment anxiety, and professional identity. Perspectives anchored in biology and behavioral genetics emphasize the influences of chromosomes, sex hormones, and brain morphology on sex differences. By contrast, social-cognitive perspectives locate the origins of these differences in socialization processes: individuals progressively acquire gender-typed behaviors by modeling same-sex others in accordance with societal expectations of gender roles (Zhou and Zhou, 2017) Previous studies have documented gender differences in social support (Xu, 2013), psychological capital (Lehoczky, 2013), employment anxiety (Xing et al., 2023), and professional identity (Han, 2014). In light of these findings, we advance the following hypothesis:

*H4*: Social support, psychological capital, employment anxiety, and professional identity differ significantly by gender.

University students confront distinct developmental tasks and environmental challenges across successive stages, and these stage-specific variations shape their psychological states and resource allocation patterns. According to the career development theory (Hu, 2008), an individual’s career development constitutes a continuous process that manifests distinct characteristics and needs at different stages. Research shows that first-year college students, having just entered the social environment, manifest relatively high professional identity (Pan, 2009), whereas fourth-year students, facing the dual pressures of a severe employment situation and limited job opportunities (Zhao and Zhang, 2008), are prone to resource depletion and consequently elevated employment anxiety. On this basis, this study proposes the following hypothesis:

*H5*: Social support, psychological capital, employment anxiety, and professional identity will differ significantly across academic years.

Self-determination theory emphasizes that human behavioral motivation is driven by three basic psychological needs: autonomy, competence, and relatedness. The degree of autonomy exercised in selecting one’s academic major is likely to shape students’ subsequent development. Research indicates that those who make autonomous decisions—motivated by intrinsic drives—are more likely to accumulate psychological capital (Li et al., 2022), develop professional identity (Chen, 2016), and proactively seek social support (Jin, 2011). In contrast, externally driven students face heightened risks of diminished interest in their major, scarce social support, weak psychological capital, and intensified employment anxiety. Accordingly, this study advances the following hypothesis:

*H6*: Significant differences exist in social support, psychological capital, employment anxiety, and professional identity according to the degree of autonomy exercised in selecting one’s academic major.

**Figure 1:**
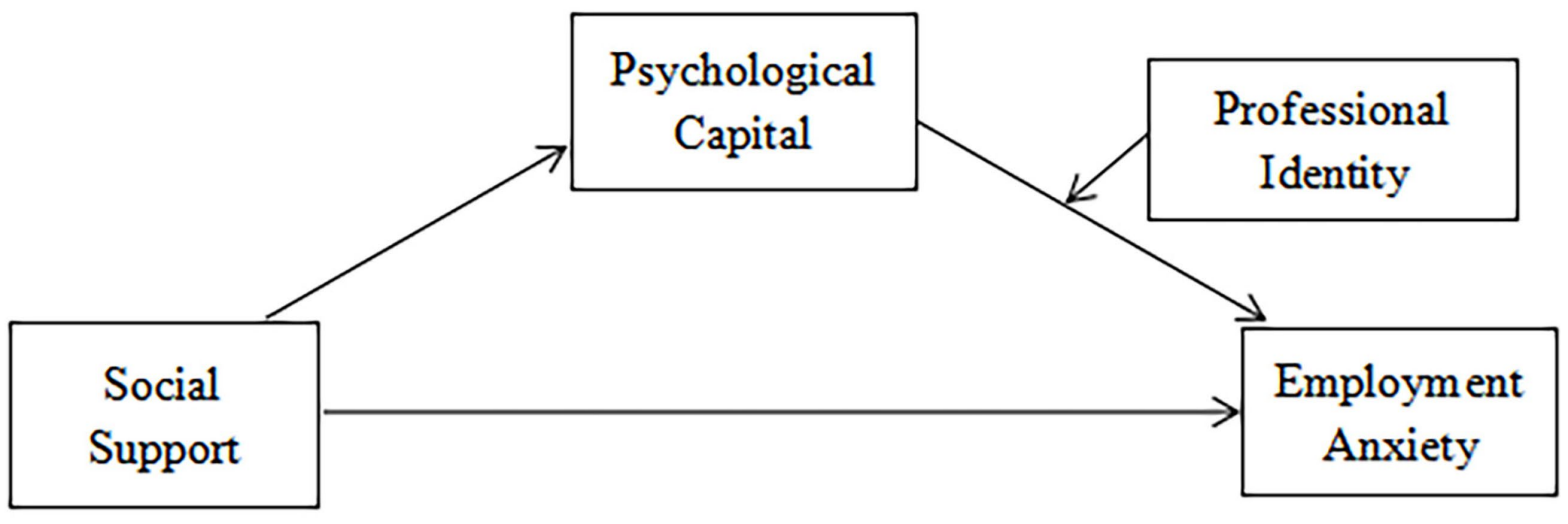
Hypothesized model.

In summary, in the current severe job market environment, preschool teacher trainees are generally troubled by employment anxiety. The employment psychological state of preschool teacher trainees is closely related to the stable development of preschool education. Therefore, it is of vital significance to deeply explore the influence mechanism of social support on employment anxiety and identify the key factors to alleviate the employment anxiety of preschool teacher trainees. At present, there is a lack of research on how internal and external factors affect employment anxiety, especially for the group of preschool teacher trainees. Therefore, this study aims to explore the relationship between social support and employment anxiety, the mediating role of psychological capital, and the moderating role of professional identity in the relationship between psychological capital and employment anxiety. The hypothesized model is shown in Figure 1.

## 2 Materials and methods

### 2.1 Research participants

To accurately understand the situation of preschool teacher trainees, this study, using a random sampling approach, recruited 1, 268 students from the preschool education major in four applied-oriented undergraduate universities in Shanghai, China. After data screening, 73 questionnaires with incomplete data were excluded, resulting in a final sample size of 1,195 students for analysis. Given the gender-structure characteristics of preschool teacher trainees, female participants in this study remarkably outnumbered males, with 1, 118 females (93.56%) and 77 males (6.44%), all aged between 18 and 22. This study administered questionnaires online via the professional survey platform Wen Juan Xing, with the participants’ informed consent. The university’s ethics committee reviewed and approved the study. The official approval number is [2024] No. 01.

### 2.2 Measures

#### 2.2.1 Social support scale

This study used Xiao Shuiyuan’s Social Support Rating Scale (Xiao, 1994). This scale has 10 items and three dimensions: subjective social support (e.g., ‘Most of my colleagues care about me”), objective social support (e.g., “When I’m in urgent need, I’ve received financial or practical help from family or friends”), and social support utilization (e.g., “When I have worries, I actively voice it to seek support and understanding”). The Social Support Rating Scale employs a four-point Likert response format; each dimension is scored by averaging its items, with higher scores reflecting greater perceived social support. The scale in this research has high reliability and validity, with a Cronbach’s *α* of 0.92 and item internal consistency above 0.89.

#### 2.2.2 Psychological capital scale

This study used the Psychological Capital Scale developed by Wang et al. (2011). It is a version revised by Wang Yanfei and other scholars based on Luthans’ scale, merging hope and optimism into one dimension to better suit Chinese college students. This scale contains 16 items across four dimensions: self-confidence (e.g., “I can state my views confidently in team activities or meetings”), optimism (e.g., “When faced with uncertainties, I usually expect the best outcome”), resilience (e.g., “Even when my work or study progress is not smooth, I will not lose heart”), and sense of responsibility (e.g., “I never shift my responsibilities to others”). The scale uses a 5-point scoring system, with higher scores indicating more positive responses. Higher scores indicate a higher level of psychological capital. In this study, the total psychological capital scale showed a high internal consistency with a Cronbach’s *α* coefficient of 0.961.

#### 2.2.3 Employment anxiety questionnaire

This study used the Employment Anxiety Questionnaire developed by Zhang and Chen (2006). This questionnaire has 26 items across four dimensions: concerns about employment prospects (e.g., “I feel anxious when I hear about the graduate employment situation in the media”), low self- confidence in job hunting (e.g., “I’m nervous facing employers’ interviews and assessments”), lack of relevant employment support (e.g., “I feel panicky due to the lack of interview performance skills”), and employment competition pressure (e.g., “I fear being rejected by employers”). The questionnaire uses a 5-point scoring system, rating from 1 (Strongly Disagree) to 5 (Strongly Agree). The overall average score represents the employment anxiety score; higher scores mean greater anxiety. In this study, the total employment anxiety scale showed a high internal consistency with a Cronbach’s *α* coefficient of 0.966.

#### 2.2.4 Professional identity scale

This study used the Professional Identity Scale for College Students developed by Pan (2009). This scale consists of 23 items across four dimensions: the cognitive dimension (e.g., “I am well informed about the employment situation of my major”), the emotional dimension (e.g., “I have positive feelings towards my major”), the behavioral dimension (e.g., “I often read books related to my major”), and the suitability dimension (e.g., “My major allows me to utilize my strengths”). The scale employs a 5-point Likert scoring system, ranging from 1 (“completely disagree”) to 5 (“completely agree”). Professional identity is assessed by the mean total score, with higher scores indicating greater professional identity. In this study, the scale showed high internal consistency with a Cronbach’s α coefficient of 0.972.

### 2.3 Demographic information

This study included the demographic information of preschool teacher trainees. Previous research indicates that students’ demographic factors relate to key variables like social support, employment anxiety, professional identity, and psychological capital. Hence, in the subsequent analysis, this study controlled for demographic variables of gender, grade, and major choice.

## 3 Research results

### 3.1 Common method bias test

This study used a questionnaire survey to collect data, which may result in common method bias. To mitigate this potential bias, Harman’s single-factor test (Matthews et al., 2006) was employed. An exploratory factor analysis without rotation was conducted, extracting three factors with eigenvalues greater than 1. The first principal component accounted for 30.516% of the variance, below the 40% threshold, suggesting the absence of substantial common method bias.

### 3.2 Descriptive and correlational analyses of study variables

As shown in Table 1, Significant gender differences were observed for social support (*p* = 0.01) and professional identity (*p* = 0.01). Specifically, male students reported higher social support (*M* = 3.80) than female students (*M* = 2.99), whereas female students reported higher professional identity (*M* = 3.79) than male students (*M* = 3.57). There are significant differences in social support (*p* = 0.003), employment anxiety (*p* = 0.019), psychological capital (*p* = 0.025), and professional identity (*p* = 0.002) across different grades. Fourth-year undergraduates (*M* = 3.24) have significantly higher employment anxiety scores than those in other grades. Freshmen report significantly higher professional identity scores (*M* = 3.82) than students in other grades. Juniors scored significantly higher than students in other grades on both social support (M = 3.06) and psychological capital (*M* = 3.79). Significant differences were found among students with different major-choice preferences in social support (*p* = 0.000), employment anxiety (*p* = 0.003), psychological capital (*p* = 0.000), and professional identity (*p* = 0.000). Specifically, students who chose their major independently have significantly higher psychological capital (*M* = 3.80), social support (*M* = 3.06), and professional identity (*M* = 3.91) scores than those who did not. Students whose major was chosen by parents or others have significantly higher employment anxiety (*M* = 3.25) than those with self-chosen majors or adjusted majors.

**Table 1:** Comparative analysis of variables across demographics ($\bar{X}\pm S$).

Demographic variables		Employment anxiety	Psychological capital	Professional identity	Social support
Gender	Male	2.99 ± 0.85	3.63 ± 0.76	3.57 ± 0.80	3.17 ± 0.51
	Female	3.16 ± 0.79	3.73 ± 0.60	3.79 ± 0.62	2.99 ± 0.49
	T	1.46	1.15	2.47*	2.55*
Grade	First year	3.12 ± 0.78	3.69 ± 0.62	3.82 ± 0.66	3.04 ± 0.48
	Second year	3.15 ± 0.78	3.63 ± 0.69	3.61 ± 0.67	2.90 ± 0.49
	Third year	3.06 ± 0.78	3.79 ± 0.56	3.80 ± 0.59	3.06 ± 0.52
	Fourth year	3.24 ± 0.80	3.73 ± 0.59	3.81 ± 0.62	2.98 ± 0.48
	F	3.34*	3.12*	4.92**	4.80**
Choice of major	Voluntary choice	3.09 ± 0.80	3.80 ± 0.59	3.91 ± 0.59	3.06 ± 0.49
	Parental or Others’ choice	3.25 ± 0.76	3.61 ± 0.62	3.59 ± 0.62	2.91 ± 0.50
	Adjustment major	3.24 ± 0.84	3.76 ± 0.56	3.54 ± 0.75	3.02 ± 0.52
	F	5.79**	15.16***	41.53***	13.43***

*n* = 1,195, *p < 0.05, **p < 0.01, ***p < 0.001.

As shown in Table 2, preschool teacher trainees’ social support (*r* = −0.34, *p* < 0.001), professional identity (*r* = −0.62, *p* < 0.001), and psychological capital are all significantly positively correlated, while social support (*r* = −0.19, *p* < 0.001), psychological capital (*r* = −0.18, *p* < 0.001), professional identity (*r* = −0.16, *p* < 0.001), and employment anxiety are all significantly negatively correlated. Additionally, there is a certain degree of correlation between grade, gender, major choice, and the main research variables, so they are included as control variables in the subsequent data analysis.

**Table 2:** Correlation analysis of variables.

Variable	1	2	3	4
1. Employment anxiety	--	−0.18***	−0.16***	−0.19***
2. Psychological capital	−0.18***	--	0.62***	0.34***
3. Professional identity	−0.16***	0.62***	--	0.28***
4. Social support	−0.19***	0.34***	0.28***	--
M	3.15	3.74	3.78	3.0
sd	0.79	0.61	0.63	0.50

*n* = 1,195, *p < 0.05, **p < 0.01, ***p < 0.001.

### 3.3 Mediation analysis of psychological capital

**Figure 2:**
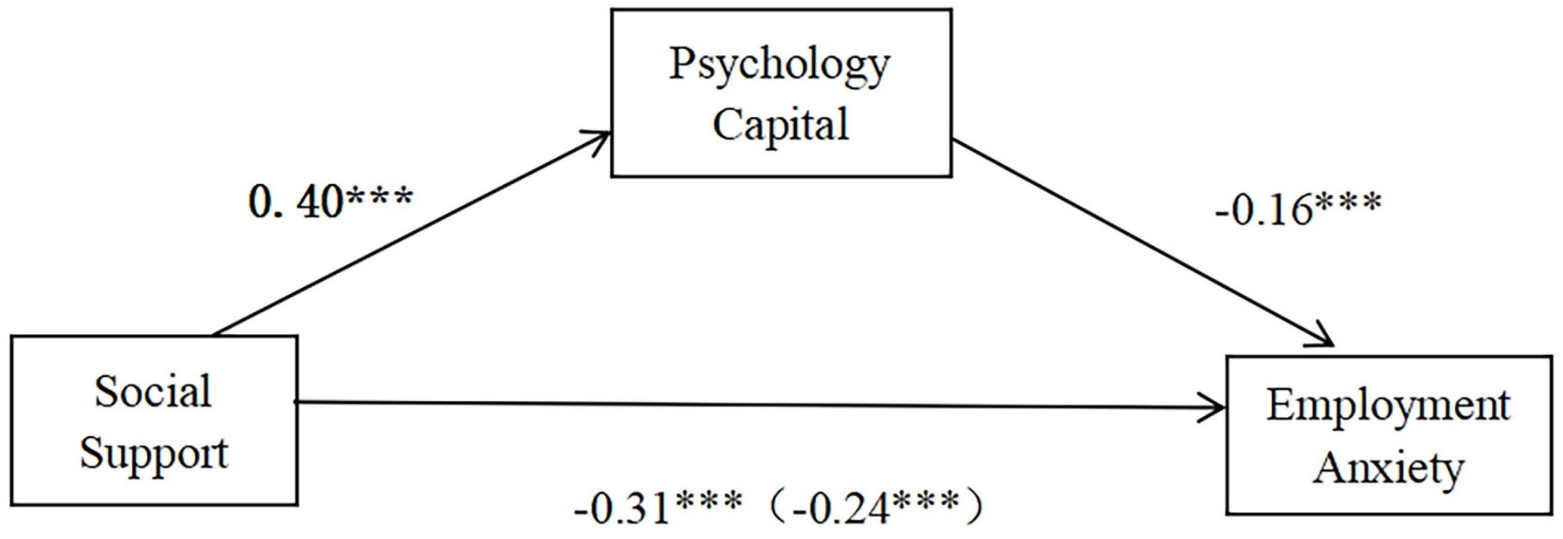
Mediating role of psychological capital, *** *p* < 0.001.

This study employed Model 4 of the SPSS PROCESS, with demographic characteristics as control variables for their potential confounding effects. As shown in Figure 2 and Table 3, social support significantly and negatively predicts employment anxiety (*β* = −0.31, *t* = −6.62, *p* < 0.001). After psychological capital was entered into the model, social support continued to exert a significant negative effect on employment anxiety (*β* = −0.24, *t* = −4.98, *p* < 0.001). Moreover, social support positively predicted psychological capital (*β* = 0.40, *t* = 11.94, *p* < 0.001), whereas psychological capital negatively predicted employment anxiety (*β* = −0.16, *t* = −4.02, *p* < 0.001), indicating that psychological capital partially mediates the relationship between social support and employment anxiety. To investigate the mediating effect, this study employed the bias-corrected bootstrap technique and performed 5,000 bootstrap samples using PROCESS. The results revealed a significant indirect effect, with the value of −0.06.

**Table 3:** Mediation model results.

Result variables	Predictor variables	*R*	*R* ^2^	*F*	*β*	*t*	95% CI	
Employment anxiety	Social Support	0.23	0.05	11.07	−0.31	−6.62***	−0.40	−0.22
	Gender				0.10	0.91	−0.11	0.31
	Grade				0.04	1.93	−0.00	0.08
	Major choice				0.09	2.10	0.01	0.17
Psychological capital	Social Support	0.38	0.15	33.79	0.40	11.94***	0.34	0.47
	Gender				0.18	2.26*	0.02	0.34
	Grade				0.03	1.92	−0.00	0.06
	Major choice				−0.10	−3.28***	−0.16	−0.04
Employment anxiety	Social Support	0.26	0.07	11.92	−0.24	−4.98***	−0.34	−0.15
	Psychological capital				−0.16	−4.02***	−0.24	−0.08
	Gender				0.13	1.18	−0.09	0.34
	Grade				0.04	2.16*	0.00	0.08
	Major choice				0.07	1.73	−0.01	0.15

*n* = 1,195, *p < 0.05, **p < 0.01, ***p < 0.001.

### 3.4 Moderated mediation analysis based on professional identity

This study examined the moderating effect of professional identity in the mediation model by employing Model 14 of PROCESS. As shown in Table 4, the interaction between psychological capital and professional identity significantly predicted employment anxiety (*β* = −0.16, *t* = −4.30, *p* < 0.001), which indicated that the moderating effect of professional identity on the relationship between psychological capital and employment anxiety was significant. In other words, the effect of preschool teacher trainees’ psychological capital on employment anxiety is contingent upon their level of professional identity.

**Table 4:** Results of the moderated mediation model.

Result variables	Predictor variables	*R*	*R* ^2^	*F*	*β*	*t*	95% CI	
Employment anxiety	Psychological capital	0.29	0.08	11.88	0.50	3.40***	0.21	0.79
	Professional identity				0.51	3.47***	0.22	0.79
	Psychological capital*Professional identity				−0.16	−4.30***	−0.24	−0.09
	Social support				−0.23	−4.81***	−0.33	−0.14
	Gender				0.12	1.04	−0.10	0.33
	Grade				0.04	2.15*	0.00	0.08
	Major choice				0.05	1.21	−0.03	0.13
Psychological capital	Social support	0.38	0.15	33.79	0.40	11.94***	0.34	0.47
	Gender				0.18	2.26*	0.02	0.34
	Grade				0.03	1.92	−0.00	0.06
	Major choice				−0.10	−3.28***	−0.16	−0.04

*n* = 1,195, *p < 0.05, **p < 0.01, ***p < 0.001.

**Figure 3:**
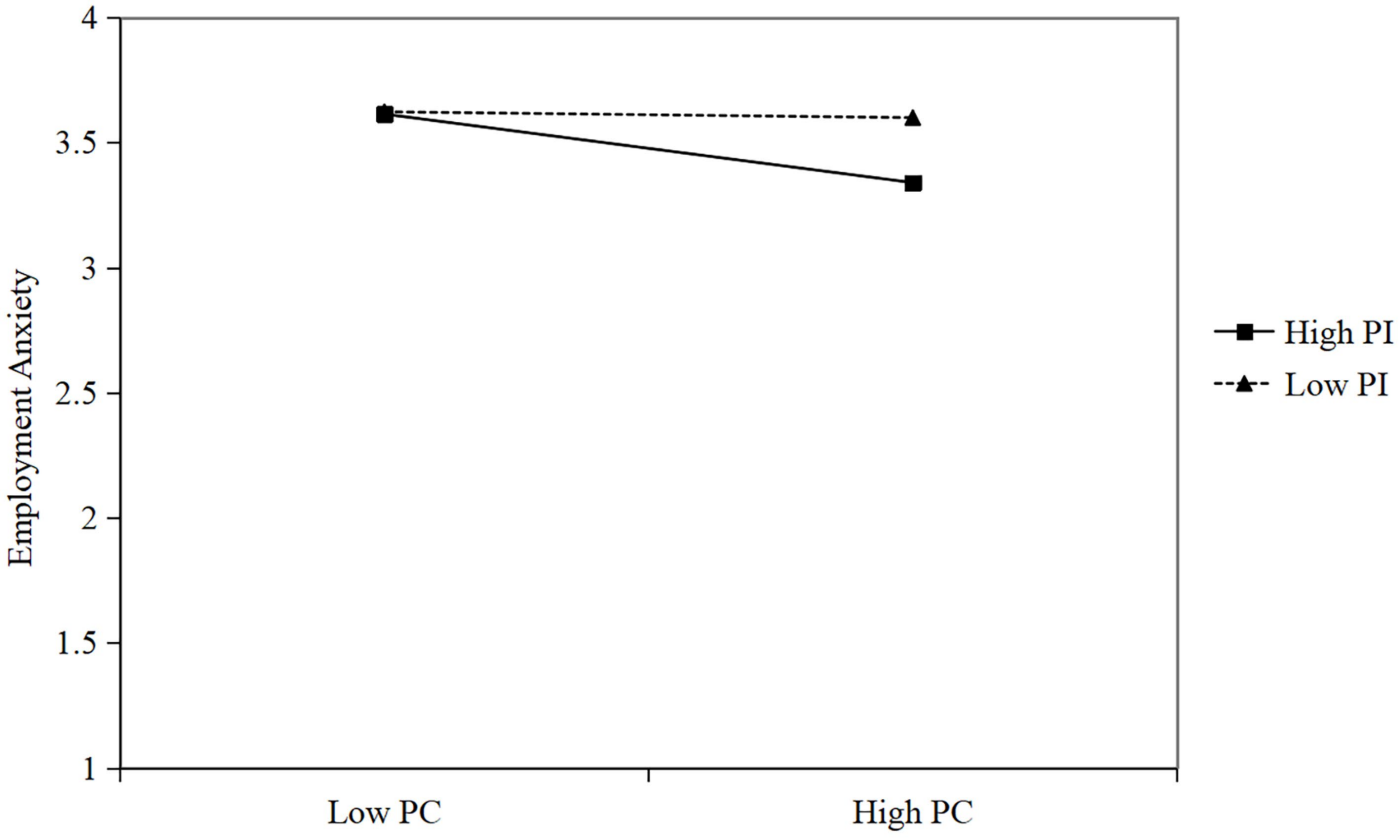
Moderating effect of professional identity on psychological capital and employment anxiety. PC, Psychological Capital; PI, Professional identity.

Furthermore, simple slope analyses were conducted to elucidate the the thematic modeling mechanism of professional identity. This study classified participants into low- and high-professional-identity groups. Simple slope analyses revealed that psychological capital exerted a significantly stronger negative effect on employment anxiety for preschool teacher trainees with high professional identity (*β* = −0.26, *t* = −4.94, *p* < 0.001) than for those with low professional identity (*β* = −0.07, *t* = −1.43, *p* > 0.05). As illustrated in Figure 3, when preschool teacher trainees reported higher levels of professional identity, psychological capital exerted a stronger effect on employment anxiety than among those with lower professional identity. Professional identity thus emerges as a pivotal factor in mitigating employment anxiety among preschool teacher trainees, enhancing the buffering effects of social support and psychological capital on employment anxiety.

## 4 Discussion

### 4.1 Demographic differences in social support, psychological capital, professional identity, and employment anxiety among preschool teacher trainees

This study revealed significant gender differences in social support and professional identity, whereas no significant gender differences were observed in psychological capital or employment anxiety; thus, Hypothesis 4 received partial support. These findings are partially consistent with prior research (Xu, 2013; Han, 2014; Shi, 2024). These findings reflect that gender-role socialization exerts differential influences on social support and professional identity. Nevertheless, the absence of gender differences in employment anxiety underscores the structural nature of contemporary societal risks. In line with Beck’s theory of the risk society (Ulrich, 1992), structural employment risks have transcended traditional gender-role buffers, engendering a “risk equalization” effect. Uncertainty in the contemporary labor market functions as a non-discriminatory stressor, exerting an equivalent impact on male and female university students and elevating employment anxiety into a trans-gender, shared stress experience. This study advances the application of risk-society theory by demonstrating that the equalizing effect of structural employment risks can override the buffering mechanisms traditionally associated with gender roles. Simultaneously, it redefines the boundaries of gender-role socialization theory: while differential gender effects persist in social support and professional identity, these effects become “disembedded” in the face of systemic risk. At the practical level, the findings suggest a dual-pathway framework for university career guidance: gender-differentiated support strategies must be implemented alongside universal intervention programs that transcend gender boundaries.

In addition, the study documented significant grade-level differences in social support, psychological capital, employment anxiety, and professional identity, thereby corroborating Hypothesis 5 and aligning with extant research (Zhang, 2023; Liu and Liu, 2007; Zhao and Zhang, 2008; Pan, 2009). This finding is consistent with the career development theory. Specifically, first-year students—having just entered a novel academic environment-experience a sense of novelty toward their field of study and display a degree of cognitive bias, which collectively yields comparatively high levels of professional identity. Developmentally, third-year students have established stable social networks and, through prior coursework, have accumulated substantial experience and skills, resulting in fully developed psychological capital. Conversely, fourth-year students confront heightened resource depletion and elevated employment pressures, precipitating declines in psychological capital and corresponding increases in anxiety. This study furnishes empirical corroboration for career development theory by confirming the age-graded characteristics of university students’ social and psychological resources, thereby deepening the theory’s explanatory framework for the dynamic nature of their career progression. Moreover, the documented grade-specific variations furnish targeted pathways for precision interventions within university mental-health and career-education systems.

Finally, the study observed significant differences in social support, psychological capital, employment anxiety, and professional identity across varying degrees of autonomy in major selection, thereby confirming Hypothesis 6 and aligning with prior research (Jin, 2011; Li et al., 2022; Chen, 2016; Yan et al., 2003). Specifically, students who chose their major autonomously exhibited significantly higher levels of psychological capital, social support, and professional identity than their counterparts in other categories, a finding consistent with self-determination theory. In line with the self-determination theory, students who autonomously choose their major satisfy their need for autonomy, activate intrinsic academic motivation, foster positive professional identity, proactively construct social-support networks, accumulate psychological capital, and maintain an optimistic outlook toward future employment. At the theoretical level, this study extends the application framework of self-determination theory within higher education by demonstrating that satisfaction of basic psychological needs—autonomy in particular—exerts sustained effects on university students’ development, thereby providing longitudinal evidence for the enduring “motivation types and mental health” linkage. At the practical level, this study furnishes key evidence for universities to protect students’ autonomy in major selection during the admissions process and to deliver professional-identity reconstruction and anxiety interventions for students who did not choose their major autonomously.

### 4.2 The effects of social support on employment anxiety among preschool teacher trainees

This study demonstrates that social support exerts a significant negative effect on employment anxiety, thereby confirming Hypothesis 1 and corroborating previous research (Fan et al., 2020). Specifically, higher levels of perceived social support are associated with lower employment anxiety. These findings are also consistent with both the main-effect and buffering-effect models of social support. The main-effect model of social support posits that social support may yield broadly beneficial outcomes (Cohen and Wills, 1985). Accordingly, preschool teacher trainees’ access to tangible support (Zhou, 2022), emotional support (Wang, 2009), and informational support can directly attenuate employment anxiety. The buffering-effect model of social support maintains that, under stressful conditions, social support mitigates the adverse impact of stressful events, thereby preserving and enhancing individuals’ psychological and physical well-being (Cohen and Wills, 1985). Thus, when preschool teacher trainees obtain effective social support, they are able to confront current challenges with a positive mindset and thereby alleviate employment-related anxiety (Yu and Cheng, 2013).

This study advances the application of social support theory to the specific population of preschool teacher trainees, enriches the theoretical framework of employment-related psychology, and furnishes empirical evidence for both the main-effect and buffering-effect mechanisms, thereby extending the applicability of the stress-buffering model within the educational domain. Simultaneously, this study elucidates the pathways through which social support influences employment anxiety, thereby furnishing a theoretical foundation and reference framework for future research. Moreover, the study provides empirical evidence and practical guidance for the coordinated construction of a social support system for preschool teacher trainees by governments, educational institutions, and families.

### 4.3 The mediating role of psychological capital between social support and employment anxiety among preschool teacher trainees

Psychological capital was found to exert a partial mediating effect between social support and employment anxiety, thereby confirming Hypothesis 2 and aligning with prior research (Cao, 2024). This finding suggests that robust social support operates as a protective mechanism: when students confront employment-related stress, they can attenuate their anxiety levels via the mediating effect of psychological capital. These findings are also consistent with COR theory, which posits that social support constitutes a valued resource for individuals, whereas psychological capital represents the outward manifestation of psychological resources (Hobfoll, 2002). Social support enhances individuals’ psychological capital; in turn, those with greater psychological capital—characterized by heightened self-efficacy (Xing et al., 2023; Li, 2021), hope, and resilience (Lian, 2022) are better equipped to confront challenges actively (Shu et al., 2021) and thereby buffer employment anxiety.

This study deepens the theoretical understanding of COR theory by explicating the social support - psychological capital - employment anxiety pathway and revealing the underlying resource-gain spiral mechanism. This finding not only enriches the theoretical connotation of psychological capital as a mediating variable, but also provides robust empirical evidence for elucidating how social support is effectively transformed into psychological capital and ultimately yields protective psychological effects that reduce employment anxiety. At the practical level, this study furnishes pivotal scientific evidence for constructing an integrated intervention model that links social support, psychological capital, and mental health.

### 4.4 The moderating role of professional identity between psychological capital and employment anxiety among preschool teacher trainees

This study found that, within the mediation model, professional identity moderates the relationship between psychological capital and employment anxiety, thereby confirming Hypothesis 3. Compared with preschool teacher trainees who report low professional identity, those with high professional identity exhibit higher psychological capital and a stronger attenuating effect on employment anxiety. High-level professional identity thereby leverages psychological capital to amplify the buffering effect of social support on employment anxiety.

COR theory posits that individuals strive to acquire, retain, and protect valued resources; as a critical psychological resource, psychological capital enables individuals to cope effectively with stress. (Hobfoll, 2002) Nevertheless, the efficacy of psychological capital is not invariant across contexts. Professional identity, conceptualized as a resource, denotes the degree to which an individual accepts and endorses his or her chosen academic major (Pan, 2009). Compared with their low-identity ones, preschool teacher trainees who possess high professional identity command solid disciplinary knowledge and skills (Wang and Wei, 2025), stronger self-efficacy (Fu and Wang, 2023), greater career adaptability (Su and Wang, 2021), and clearer employment expectations (Song et al., 2024); consequently, they experience markedly lower employment anxiety. Conversely, individuals with low professional identity report diminished academic self-efficacy and heightened employment pressure (Song et al., 2024); consequently, even when endowed with relatively high psychological capital, they struggle to translate it into effective capacities for meeting employment challenges. This study extends the application scope of COR theory and elucidates the pivotal role of resource synergy. Specifically, the study delineates professional identity as a critical boundary condition and clarifies its moderating mechanism in determining the efficacy of psychological capital in alleviating employment anxiety. This finding enriches the theoretical framework of moderated mediation by opening a new perspective on the complex interplay between professional identity and psychological capital. At the practical level, the findings provide an empirical basis for universities to implement precise and differentiated employment-related psychological interventions, underscoring the centrality of cultivating professional identity.

## 5 Educational recommendations

In the context of the continuous decline in population birth rate, preschool teacher trainees face numerous employment difficulties and challenges, such as intense job competition and insufficient job positions. These issues not only affect students’ personal career development but also influence the healthy development of early childhood education and social progress. Based on the above findings, this study proposes the following educational recommendations.

### 5.1 Optimizing the social support system

Optimize the social support system and foster synergistic talent cultivation among multiple stakeholders. The family environment, particularly parents’ parenting styles, is crucial to psychological development. Therefore, parents should proactively improve inappropriate educational methods and provide strong support for college students’ healthy growth and employment. Society should create a fair and tolerant employment environment, reduce restrictions on new graduates, eliminate gender discrimination and the parochial notion of “prestige - school advantage,” use ability as the criterion, and provide students with more opportunities for fair competition (Zhang, 2005). Colleges and universities should attach great importance to employment-related psychological counseling. They need to carry out a correct analysis of the employment situation and improve the functions of employment centers. These centers should play an active role in career guidance, providing employment information, exploring job opportunities, and conducting employment assessments. Meanwhile, relevant government departments should also provide more support and assistance to college students through legal systems and policy measures.

### 5.2 Enhancing students’ professional identity

As a key motivational determinant, professional identity directly influences preschool teacher trainees’ expectations and ultimate decisions to enter the preschool teaching workforce (Liu, 2014). First, the structure of the pre-school teacher-education major must be optimized and adjusted. A pronounced supply-demand imbalance exposes preschool teacher trainees to the risk of structural unemployment, thereby eroding their professional identity. In the context of persistently declining birth rates, universities should dynamically adjust the scale and curricular architecture of the major, refining course offerings to enhance the alignment between graduate supply and labor-market needs. Second, the learning environment should be substantially enhanced. Universities are advised to upgrade pre-school education facilities, expand the availability of discipline-specific learning resources, and increase students’ access to authentic practice opportunities, thereby reinforcing their behavioral identification with the profession. Concurrently, universities should regularly convene academic conferences and specialized lectures, cultivating a rich intellectual climate that keeps students informed of disciplinary frontiers and career prospects, thereby reinforcing their professional confidence and deepening their affective commitment to the field. Finally, institutions should implement tiered and progressive intervention strategies tailored to students’ varying levels of professional identity. For students exhibiting high professional identity, interventions should focus on leveraging their psychological capital into core employability competencies, whereas for those with low professional identity, priority should be given to consolidating their sense of professional belonging and enhancing their capacity to acquire essential resources.

### 5.3 Implementing career planning education

Colleges and universities should promptly guide students in career planning, helping them accurately understand current employment conditions, clarify their self- positioning, set reasonable job-hunting goals, and maintain a positive job-hunting attitude. Firstly, career planning education should span the entire university education process, offering more targeted programs to students of different years and majors (Xu, 2013). Secondly, it is essential to establish a career development curriculum system, campus culture, specialized guidance and consulting services, and utilize alumni resources to conduct career planning education. This integrated approach aims to achieve the best results (Xu, 2016). Thirdly, there should be a focus on cultivating and accumulating key resources such as individuals’ positive psychological capital. This involves building up their confidence for employment and motivating them to actively invest in resources to enhance their personal competitiveness (Shi et al., 2023).

## 6 Conclusion and research limitations

This study explores the association between social support and employment anxiety, as well as the mediating role of psychological capital and the moderating role of professional identity. The study reveals partial significant differences in social support, psychological capital, employment anxiety, and professional identity across gender, grade level, and major choice. Social support not only directly and negatively predicts the degree of employment anxiety but also alleviates employment anxiety through psychological capital. Meanwhile, professional identity positively moderates the relationship between psychological capital and employment anxiety. These findings offer vital insights into promoting college students’ employment and alleviating their employment anxiety.

This study has certain limitations. Firstly, the sample size was relatively small and confined to preschool teacher trainees in Shanghai. Thus, the generalizability of the findings requires further validation. Future research is recommended to expand the sample size and diversify the participant population. Secondly, Owing to the marked gender imbalance within the pre-school education major, female students constitute the overwhelming majority in the present sample; consequently, gender comparisons lack representativeness. Thirdly, this study adopted a cross-sectional research design. For future research, it is recommended to employ a longitudinal research method to more thoroughly investigate the causal relationships between variables. Lastly, while this study confirmed the relationship between social support and employment anxiety, as well as the roles of psychological capital and professional identity, employment anxiety may also be shaped by academic factors (e.g., academic stress) and familial factors (e.g., parental expectations). Future research should therefore incorporate these variables into the model to elucidate their underlying mechanisms.

## Data Availability

The raw data supporting the conclusions of this article will be made available by the authors, without undue reservation.
